# A Novel Multiplayer Screen-Based Simulation Experience for African Learners Improved Confidence in Management of Postpartum Hemorrhage

**DOI:** 10.3389/fpubh.2017.00248

**Published:** 2017-09-26

**Authors:** Jeffrey M. Taekman, Megan F. Foureman, Fred Bulamba, Michael Steele, Emily Comstock, Andrew Kintu, Amy Mauritz, Adeyemi Olufolabi

**Affiliations:** ^1^Department of Anesthesiology, Duke University School of Medicine, Durham, NC, United States; ^2^Duke University Medical Center, Durham, NC, United States; ^3^Duke University School of Medicine, Durham, NC, United States; ^4^Department of Anesthesia, Makerere University College of Health Sciences, Kampala, Uganda; ^5^Duke University School of Nursing, Durham, NC, United States

**Keywords:** distance education, educational technology, medical education, serious games, simulation

## Abstract

**Introduction:**

Postpartum hemorrhage (PPH) remains a global challenge, affecting thirteen million women each year. In addition, PPH is a leading cause of maternal mortality in Asia and Africa. In the U.S.A., care of critically ill patients is often practiced using mannequin-based simulation. Mannequin-based simulation presents challenges in global health, particularly in low- or middle-income countries. We developed a novel multiplayer screen-based simulation in a virtual world enabling the practice of team coordination with PPH. We used this simulation with learners in Mulago, Uganda. We hypothesized that a multiplayer screen-based simulation experience would increase learner confidence in their ability to manage PPH.

**Methods:**

The study design was a simple pre- and a post-intervention survey. Forty-eight interprofessional subjects participated in one of nine 1-h simulation sessions using the PPH software. A fifteen-question self-assessment administered before and after the intervention was designed to probe the areas of learning as defined by Bloom and Krathwohl: affective, cognitive, and psychomotor.

**Results:**

Combined confidence scores increased significantly overall following the simulation experience and individually in each of the three categories of Bloom’s Taxonomy: affective, cognitive, and psychomotor.

**Conclusion:**

We provide preliminary evidence that multiplayer screen-based simulation represents a scalable, distributable form of learning that may be used effectively in global health education and training. Interestingly, despite our intervention being screen-based, our subjects showed improved confidence in their ability to perform psychomotor tasks. Although there is precedent for mental rehearsal improving performance, further research is needed to understand this finding.

## Introduction

Despite improvements in the rate of maternal mortality worldwide, more than 250,000 women die each year during the perinatal period (1), a good number from postpartum hemorrhage (PPH). PPH remains a global challenge, affecting thirteen million women each year (2) and is a leading cause of maternal mortality in Asia and Africa (3).

Treating PPH requires two critical steps: recognition of the event and an appropriate clinical and timely management of bleeding (3). In low- and middle-income countries (LMIC), failing either of these steps often leads to maternal death. A great deal of PPH management involves teamwork and communication. Thus, teamwork and communication training is a critical component in the management of any life-threatening event.

Communication failures are responsible for approximately two-thirds of iatrogenic harm in healthcare (4). Over the last decade, in the United States (U.S.), teamwork and communication has been recognized as a critical component of clinical management, largely in response to the Institute of Medicine’s (IOM) To Err is Human (5). However, learning to function as a team takes practice (4). In response to the call to action by the IOM, the U.S. Department of Defense and the Agency for Healthcare Research and Quality developed TeamSTEPPS, “a flexible, evidence-based toolkit to improve patient safety through enhanced communication and other teamwork skills.” (6) The TeamSTEPPS curriculum leverages two decades of experience with teamwork and communication in high-risk industries. These materials are freely distributed over the internet and can be customized to any healthcare setting.

Over the past two decades, there has been explosive growth in the use of mannequin-based simulation in healthcare education and training (7). Teamwork and communication is often taught using mannequin-based simulation. High-fidelity, mannequin-based simulation recreates an environment where learners manage realistic patients in context (8). Students practice their profession in scripted scenarios designed to challenge clinical decision-making, critical thinking, and teamwork. What makes simulation so effective, in all of its forms, is it allows learners to practice in the context of how the learning will be applied, a construct called situated cognition (9). Simulation has many advantages over lecture-based education, including interactivity, reproducibility, and the ability to allow learners to practice in teams and in context (8).

Simulations, both high and low fidelity, are effective training methods for obstetric emergencies (10). Although mannequin-based simulation is an effective high-fidelity training method, it has limitations in global health. Mannequin-based simulation, when used correctly, requires expensive equipment, a complex video and computer infrastructure, and dedicated, specially trained personnel. Other major limitations include the requirement for individuals to co-locate and the difficulty scaling training to a large learner population (8). Moving simulation equipment internationally to LMICs adds significantly to the logistical complexity and expense of training.

More recently, largely due to the increase in power of computers, increasing speed of the internet, and capabilities of software, screen-based simulation has become more common in healthcare education (8, 11). Some screen-based simulation leverages commercial gaming technology. Screen-based simulation offers many advantages over mannequin-based simulation, especially for global health. These advantages include the ability to run software on relatively inexpensive computers, the ability to easily distribute and scale, and may be accessed regardless of the learner’s location (8). In addition, because screen-based simulation takes place on a computer over a network, every decision and action taken by the learner becomes a data point, enabling analysis through learning analytics (big data for education) (11).

**Figure 1:**
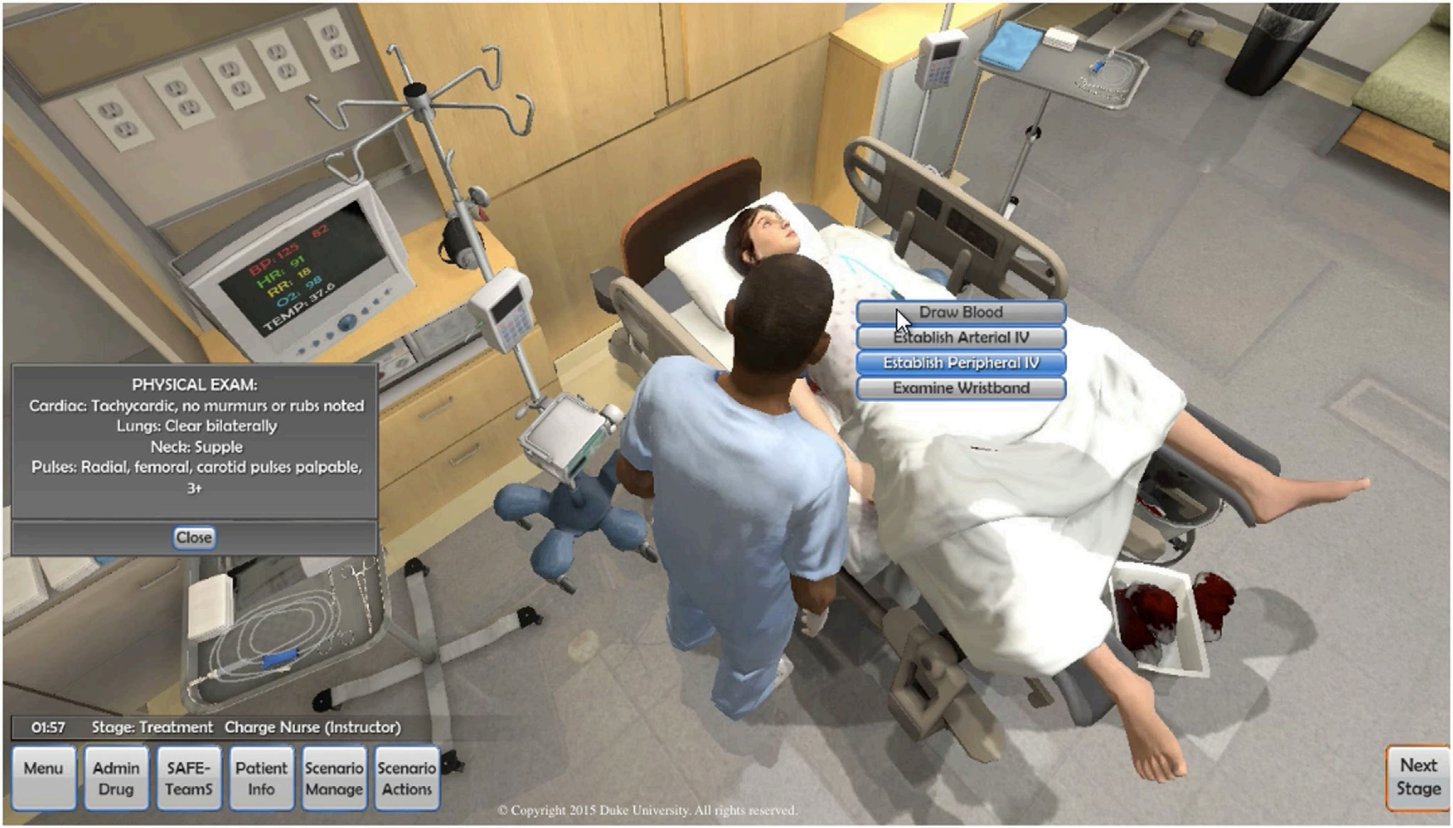
Screen-shot of 3DiTeams—postpartum hemorrhage—multiplayer screen-based simulation. Each character is controlled by a unique individual using a computer, mouse, and voice-over-IP headset.

In collaboration with a local government contractor (Applied Research Associates, Cary, NC, USA), we developed a multiplayer screen-based simulation that enables the practice of teamwork and communication (using the TeamSTEPPS) in the setting of PPH. The software is one of several packages under the Immersive Learning Environments @ Duke (ILE@D) Umbrella (http://lr.simcenter.duke.edu/)—The PPH software enables interprofessional learners, regardless of their location in the world, to share a virtual birthing room and care for a virtual patient (Figure 1). Simulation with the software is similar to that of mannequin-based simulation in that learners participate in the virtual care of a patient, then reflect on their actions during a formal debrief. These debriefs may be held locally or may leverage digital technology (e.g., Skype) to conduct the discussion at a distance.

We undertook a proof-of-concept training program using our software for PPH education in Uganda. We *hypothesized* that our multiplayer screen-based simulation would be effective in increasing learner confidence in the management of PPH.

## Materials and Methods

### Simulation

In Spring 2015, we conducted nine 1-hour sessions over a 5-day period. Due to the educational nature of this study, it was not registered in a clinical trial registry. Following IRB approval and written informed consent, 48 subjects were recruited through flyers placed at Mulago Hospital/Makerere University in Mulago, Uganda. Two to ten interprofessional participants were present for each session. Learners were trainees or professionals from: Obstetrics, Midwifery, and Anesthesiology.

Our software, built on the Epic Games platform (Cary, NC, USA). Epic is the technology behind many commercial video games. Epic runs on inexpensive, easily accessible computers with internet connections. In our case, the Epic technology was repurposed to develop a healthcare learning activity. The simulation was customized to reflect available resources in Uganda (e.g., whole blood only instead of multiple blood fractions). Learners participated in the simulation from individual computers, controlling the actions of their character using a computer mouse. Voice over IP (VOIP) enables audio communication over the internet. Participants wore VOIP headsets with microphones in order to facilitate teamwork and communication.

After a brief introduction to the project, each learner practiced moving their avatar in the virtual environment, interacting with equipment, the patient, and other learners. In addition, each participant practiced administering drugs as well as verifying and administering blood products. Following this introductory period, the simulation was re-initiated for the actual learning experience.

Learners participated in the same, instructor lead simulation experience focused on PPH. The simulation experience consisted of a 30-min screen-based facilitator-lead simulation followed by a 30-min debrief with faculty both in Uganda and in the United States. The participants communicated with distant faculty using VOIP headsets.

The sessions in Uganda were led by a trained simulation facilitator from the Duke University Human Simulation and Patient Safety Center. Team members in the US were able to watch, listen, and participate in the simulation in real time. For the debrief, the facilitator in Uganda was joined in the debrief by team members in the US *via* Skype (Microsoft, Redmond, WA, USA).

### Study

The study design was a pre- and a post-intervention survey. In addition to demographic information, questions to probe the learners’ familiarity with computers and computer games were included.

A fifteen-question self-assessment (Appendix A in Supplementary Material) was designed to probe the three areas of learning as defined by Bloom and Krathwohl: psychomotor, cognitive, and affective (12). The questionnaire consisted of four psychomotor questions, six cognitive questions, and five affective questions targeted at management of PPH. The questionnaire used a scale from 0 (not confident) to 10 (very confident). Each participant completed the questionnaire before and after their simulation experience.

The confidence questions (Appendix A in Supplementary Material) were meant to reflect representative tasks of management of PPH. Affective tasks included such items as comfort with the obstetric environment, and the ability to communicate effectively with team members. Cognitive tasks included recognizing and treating PPH, timing and administration of drugs, and correct responses to alterations in patient condition. Psychomotor tasks included performance of fundal massage, placement of intravenous lines, and administration of medications *via* the correct route.

In addition to post-intervention confidence questions, the learners answered a series of questions to gage their immersion in the training, the time spent in the simulation, and their thoughts about screen-based simulation.

Data were collected on paper by trainers before and immediately after the intervention. Confidence data, pre- and post-simulation, were compared using a paired *t*-test comparison. Comparison data are reported as mean ± SD.

## Results

Learners were 60% male and 40% female (See Table 1). Age range was 21–55 years with an average age of 31.2 years. Learners’ professions were 10% Obstetrics, 15% Midwifery, 48% Anesthetic Officers, and 27% Anesthesiologists. Midwives, not nurses, participate in live births at Makerere University Hospital.

**Table 1:** General demographics of subjects.

Age range (years)	21–55
Average age (years)	31.2
Median age (years)	29
**Gender**	
Male	60%
Female	40%
Obstetric experience	96%
Mannequin sim experience	45%
Screen-Based sim experience	15%
**Profession**	
Obstetrician	10%
Midwife	15%
Anesthetic Officer	48%
Anesthesiologist	27%

Ninety-six percent of learners had at least some experience on the obstetrics ward. Fifty-six percent of learners had little or no experience working with computers. Although 46% of learners had participated in a mannequin-based simulation, only 15% had experienced a computer-based simulation. Despite the limited exposure prior to our intervention, 87% believed screen-based simulation would be an effective way to learn the skills of caring for a woman with PPH. Eighty-two percent of learners played 2 hours or less a week of video games. Ninety-six percent of the participants had never played First Person Shooter games (the software platform the simulation is built upon).

**Figure 2:**
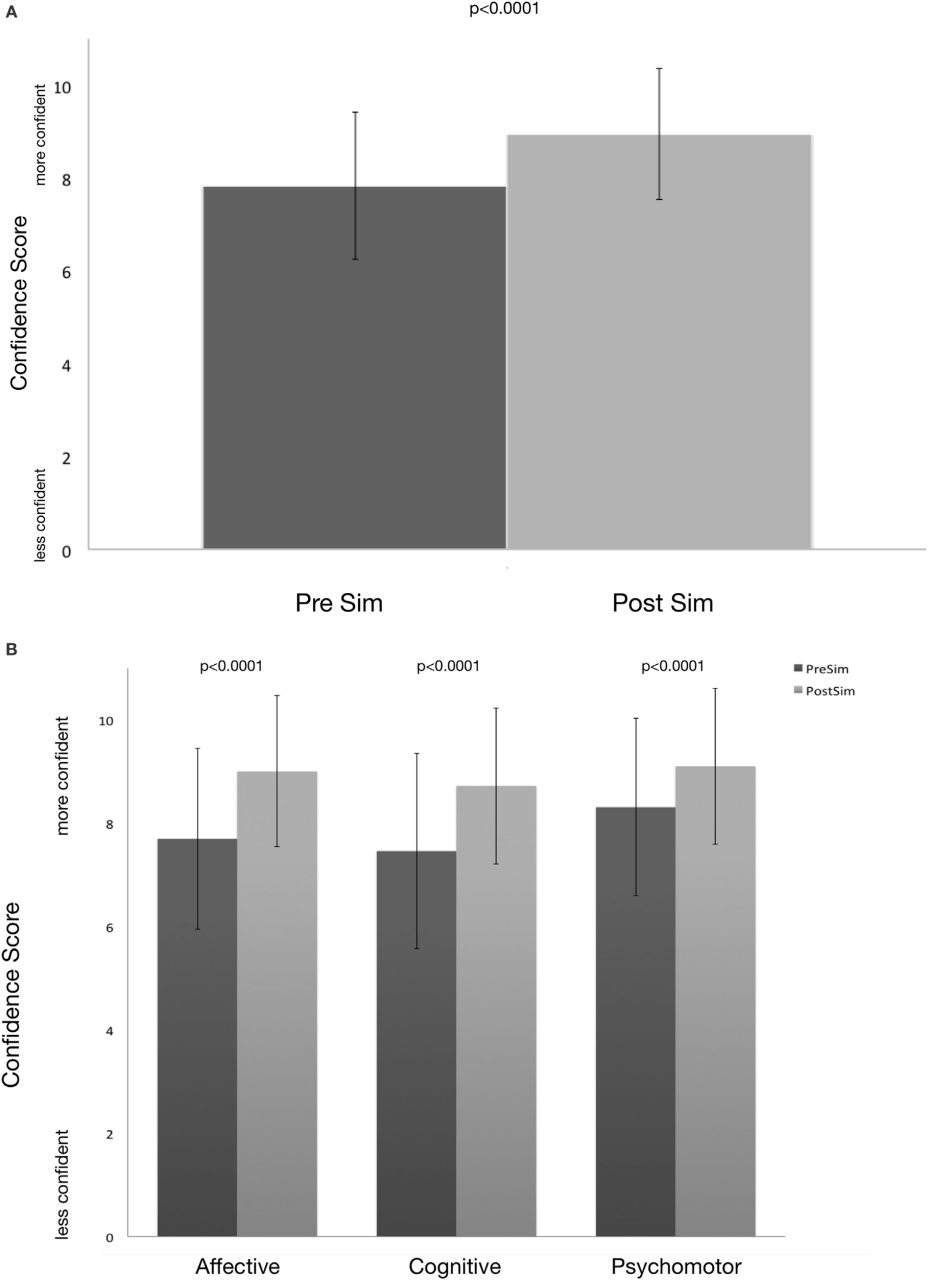
**(A,B)** Pre- and post-learner confidence in management of postpartum hemorrhage (value ± S.D^.^) Questions were categorized into one of three domains of Bloom’s Taxonomy. Pre and post survey consisted of four psychomotor questions, six cognitive questions, and five affective questions. Following the screen-based simulation experience, significant gains in confidence were seen in each of the domains **(B)** as well as the combined overall confidence score **(A)**.

Self-reported confidence scores increased significantly overall following the simulation experience (pre = 7.83 ± 1.55, post = 8.95 ± 1.42, *p* < 0.001) (Figure 2A and Table 2) and individually in each of the three categories of Bloom’s Taxonomy: affective (pre = 7.70 ± 1.75, post = 9.00 ± 1.46, *p* < 0.001), psychomotor (pre = 8.31 ± 1.71, post = 9.11 ± 1.51, *p* < 0.001), and cognitive (pre = 7.46 ± 1.89, post = 8.73 ± 1.51, *p* < 0.001) (Figure 2B and Table 2).

**Table 2:** Pre and post learner confidence in management of postpartum hemorrhage (value ± SD).

Variable	Pre	Post	*p*
Affective	7.70 ± 1.75	9.00 ± 1.46	<0.0001
Cognitive	7.46 ± 1.89	8.73 ± 1.51	<0.0001
Psychomotor	8.31 ± 1.72	9.11 ± 1.51	<0.0001
Ave. combined	7.83 ± 1.58	8.95 ± 1.42	<0.0001

*Questions were categorized into one of three domains of Bloom’s Taxonomy. Pre and post survey consisted of four psychomotor questions, six cognitive questions, and five affective questions. Following the screen-based simulation experience, significant gains in confidence were seen in each of the domains as well as the combined overall confidence score*.

Following the simulation and debrief, 100% of learners felt screen-based simulation was an effective way to learn to care for a woman suffering from PPH. Ninety-eight percent of participants would recommend the simulation as a way to learn about PPH.

Following the simulation, learners were asked various questions about the software. Ninety-six percent of learners agreed or strongly agreed with the statement, “It is important to have access 24 hours a day, 7 days a week, 365 days a year.” Eighty-four percent agreed or strongly agreed with the statement, “I would like to participate in the simulation on my own device (rather than being required to use a dedicated computer).” Learners were mixed in their interest in having collaborators in the US—23% disagreed or strongly disagreed this was necessary, 52% agreed or strongly agreed, and 23% were neutral.

Learners valued the multiplayer aspect of the simulation. When asked if they would like to learn independently, without the need for other learners, 63% disagreed or strongly disagreed, 20% agreed, and 17% were neutral. When asked if they would like to continue to learn in an environment with other human team players, 98% agreed and 2% were neutral.

The learners valued feedback, both in-game and by a live facilitator. When asked if it is important to have immediate, in-game feedback, 91% agreed, 2% disagreed, and 7% were neutral. When asked if it is important to have feedback in a debrief with a live facilitator, 94% agreed or strongly agreed, 2% disagreed, and 4% were neutral.

## Discussion

This is the first description of a multiplayer screen-based simulation/virtual environment used in global health. Our study with learners in Uganda, using our novel software as the centerpiece of a simulation experience, demonstrated an increase in participants’ confidence in the management of PPH. In addition, there was wide consensus that screen-based simulation was an effective way to practice the teamwork needed for proper management of PPH. Confidence improved in all three domains of Bloom’s Taxonomy: affective, cognitive, and psychomotor. Since learners do not perform any physical procedure in screen-based simulation, a counterintuitive finding was a gain in psychomotor confidence following participation in the session.

Simulation provides an opportunity for mental rehearsal and reflection. Although more research is needed to understand how increased confidence translates into real-world performance, there is precedent for mental rehearsal improving performance in sports (13) and medicine (14). Mental reflection is also a key component in the development of expertise (15). The initial implication is that screen-based simulation, despite lacking a psychomotor component, may be a viable substitute for at least a subset of mannequin-based simulation activities. It must be pointed out that the primary focus of our simulation was teamwork and communication and only included simple psychomotor tasks. Screen-based simulation might not have been as effective in changing psychomotor confidence with more complex psychomotor tasks such as a surgical procedure. More research is needed to understand the impact of screen-based simulation on the acquisition, performance, and mastery of affective, cognitive, and psychomotor skills in the real world, both simple and complex.

**Figure 3:**
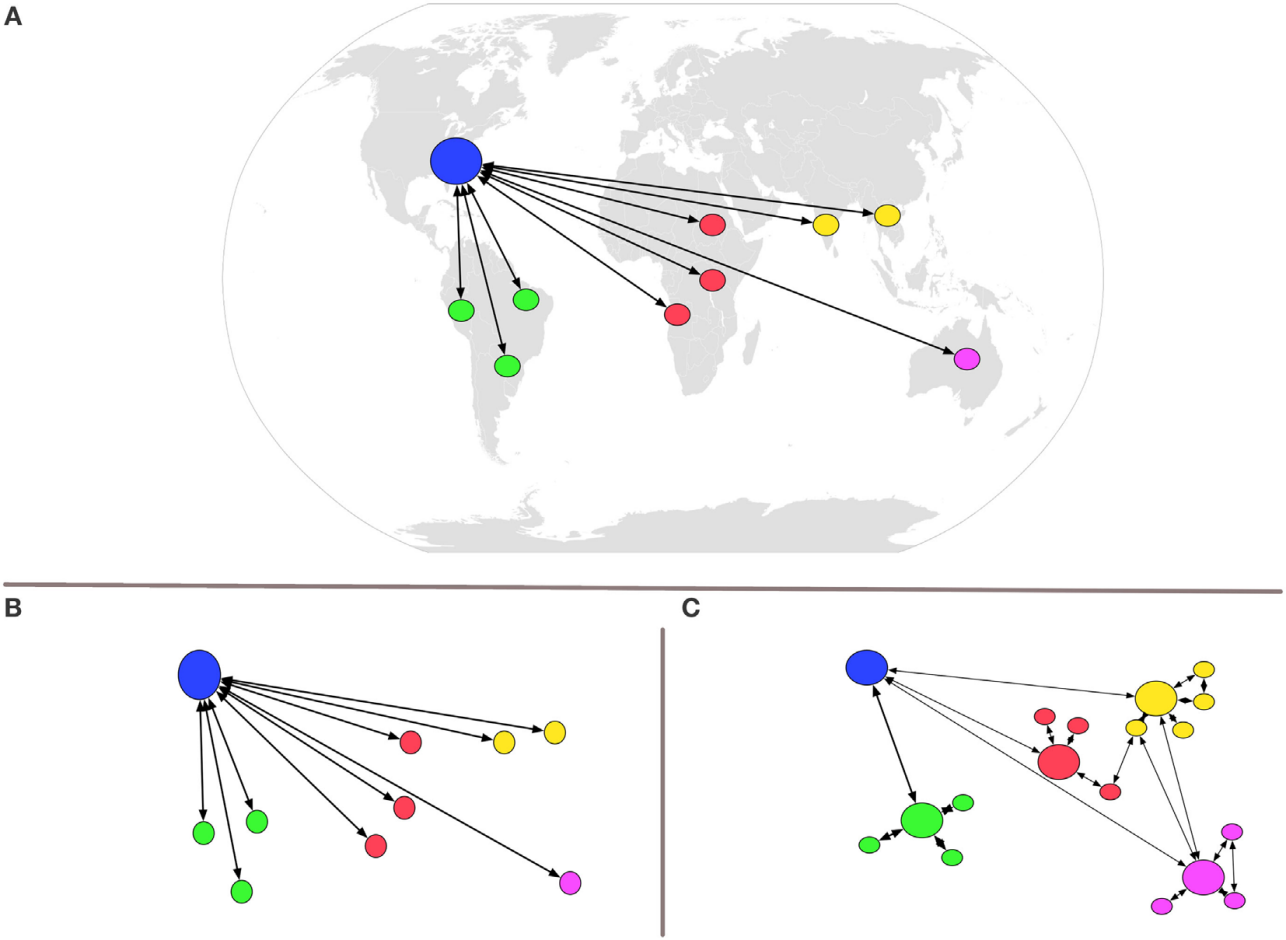
Visual representation of new opportunities enabled by multiplayer screen-based simulation. Because the software runs on readily available computers, screen-based simulation enables new opportunities for global health, including just-in-time education from the USA to anywhere **(A,B)** or anywhere to anywhere **(C)**. Configurations such as those noted in Figure 3C, since they require no more than a computer and Voice-over-IP headset, may be scaled to a global audience relatively quickly.

Screen-based simulation is infinitely more scalable and portable than mannequin-based simulation (8, 11) and offers many other advantages and opportunities for global health. These advantages include convenience for the learners and facilitators, ubiquity of equipment to run software, and overall lower cost. Because the software runs on readily available computers, screen-based simulation enables new opportunities for global health, including just-in-time education from a distant partner (USA) to anywhere (Figures 3A,B) or anywhere to anywhere (Figure 3C). Configurations such as those noted in Figure 3C, since they require no more than a computer and VOIP headset, may be scaled to a global audience relatively quickly. Furthermore, the interconnectedness of the internet can form a network of educators and learners that may link with one another repeatedly, regardless of location. The interconnectedness of this learning network enables a wide array of new learning activities and data opportunities. Marrying the data generated from our simulation with other data sources could usher in a brand new area of investigation for Global Health: Learning Analytics.

It should be noted that although only a portion of our learners had experience with gaming, they universally found the screen-based simulation useful. Although not reflected in our data, one of the common comments was how individual learners enjoyed the interprofessional nature of the exercise. Many cited interprofessional education as the most valuable part of the entire experience. Mannequin-based simulation is known to be an effective method of training teams. It appears, from our results, that screen-based simulation may be equally effective.

Our study has several limitations. Participants self-selected and, thus, may not be a truly representative sample of providers in Uganda. Furthermore, one must consider the simulation experience may not be directly responsible for the gains in learner confidence. Instead, learner improvements may have been a result of prolonged reflection on their performance. Even if this is the case, the PPH software offers a viable alternative to the way most learners in LMICs gain experience: with live (and sometimes critically ill) patients. Our PPH software set the stage for situated cognition (9)—placing the learner in the clinical environment—without risk to patients.

The original software was designed for learners in a highly resourced medical center environment. The virtual environment setting—a state-of-the-art birthing room—would be unfamiliar to learners in LMICs. Despite these incongruities in appearance, the software was effective in changing learner confidence although it is unknown what the impact of these incongruences might be.

Although our findings are promising, our results may not be generalized to other LMICs. Each country (and sometimes even hospital) has its own unique culture, hierarchy, and patient-care challenges. The challenges seen in other locations may be less amenable to change by our software. Further research is needed to understand how our findings generalize to other settings.

Self-reported outcomes have their own limitations. Ultimately educators and administrators want proof that screen-based-simulation impacts patient outcomes. Much as with the case of mannequin-based simulation, definitive proof with patient outcomes may never be possible, given the relatively low frequency and unexpected nature of critical events (16).

## Conclusion

In this report, we provide preliminary evidence that multiplayer screen-based simulation may be used effectively in global health education/clinical training. We foresee screen-based simulation ushering in a new generation of opportunities in global health education.

## Ethics Statement

This study was approved by the Duke University Medical Center IRB Department. All subjects were properly consented before participating in the study. Please see contact information below for Duke University IRB Office. Duke University IRB Office Hock Plaza, Suite 405 2424 Erwin Road Campus Box #2712 Durham, NC 27705 (919) 668-5111.

## Author Contributions

JT: designed the study, conducted the study, analyzed and interpreted the data, and prepared the manuscript. MF: helped design the study, conducted the study, collected the data, confirmed analysis, and prepared the manuscript. FB, MS, EC, and AK: conducted the study, collected data, and prepared the manuscript. AM: conducted the study and prepared the manuscript. AO: conducted the study, collected data, and prepared the manuscript.

## Conflict of Interest Statement

JT has proprietary interest in the simulation platform should it be commercialized.
