# Supplementary material for: A Novel Multiplayer Screen-Based Simulation Experience for African Learners Improved Confidence in Management of Postpartum Hemorrhage
Source: Front Public Health. 2017 Sep 26;5:248. doi: 10.3389/fpubh.2017.00248 (PMC5623004; doi:10.3389/fpubh.2017.00248)
Supplement: Supplementary file 1 [file data_sheet_1.docx]

**APPENDIX A**

**PRE SIMULATION SURVEY**

**Pre-Simulation Questions:**

1. Initials: _____

2. Age: _____

3. Sex:

a. Male

b. Female

4. Do you have experience in the obstetrics ward?

a. If yes, how many years?

5. How much experience do you have working with computers?

a. None (I have never worked with a computer or I would not be able to power on the computer and open a document by myself)

b. Small amount (I am familiar with the basics of powering on computer and logging in. I can access my email or social media from a computer)

c. Moderate amount (I can trouble-shoot minor problems on a computer. I use a computer several times a week. I feel comfortable using the computer for communication and for obtaining information)

d. Much experience (My colleagues and friends come to me for help with their computer problems. I would be uncomfortable if I did not have regular access to a computer)

6. Have you ever participated in computer-based simulation before?

a. Yes

b. No

7. Have you ever participated in mannequin-based simulation before?

a. Yes

b. No

8. Do you think mannequin based simulation is an effective way to learn the skills of caring for a woman with postpartum hemorrhage?

a. Yes

b. No

9. Do you think screen based simulation is an effective way to learn the skills of caring for a woman with postpartum hemorrhage?

a. Yes

b. No

10. How many hours a week do you play computer games?

a. None

b. 1-2 hours

c. 2-5 hours

d. 5-20 hours

e. 20+ hours

11. How many hours a week do you play FIRST PERSON SHOOTER video games?

a. None

b. 1-2 hours

c. 2-5 hours

d. 5-20 hours

e. 20+ hours

12. To what profession do you belong?

a. Nursing

b. Obstetrics

c. Midwifery

d. Anesthetic Officer

e. Anesthesiologist

Below is a series of questions relating to your comfort level with a variety of activities. Please mark the number corresponding to your comfort level on a scale of 0 to 10 (0= very uncomfortable, 5= neutral, 10=very comfortable).

1. How confident are you in the obstetrics ward? (A)

2. How confident are you in being able to work as a team? (A)

3. During the management of postpartum hemorrhage, how confident are you in your ability to delegate tasks? (A)

4. When things are not going well on the obstetric ward, how confident are you in your ability to speak up? (A)

5. When dealing with postpartum hemorrhage, how confident are you in your ability to act on directions from other members of healthcare team? (A)

6. How confident are you in your ability to perform fundal massage? (P)

7. During postpartum hemorrhage, how confident are you in your ability to place additional intravenous lines? (P)

8. During postpartum hemorrhage, how confident are you in your ability to administer intravenous medications? (P)

9. During postpartum hemorrhage, how confident are you in your ability to administer intramuscular medications? (P)

10. How confident are you in your ability to verify correct blood products before administration? (C)

11. How confident are you in your ability to recognize obstetric hemorrhage? (C)

12. How confident are you in your ability to correctly treat postpartum hemorrhage? (C)

13. In order to manage postpartum hemorrhage, how confident are you in your ability to choose appropriate drugs? (C)

14. In order to manage postpartum hemorrhage, how confident are you in your ability to recall dosages and routes for drug administration? (C)

15. During postpartum hemorrhage, how confident are you in your ability to estimate blood loss? (C)

(A)=Affective, (P)=Psychomotor, (C)=Cognitive

**APPENDIX B**

**POST SIMULATION SURVEY**

**Post-Simulation Questions:**

1. Initials: _____

Below is a series of questions relating to your comfort level with a variety of activities. Please mark the number corresponding to your comfort level on a scale of 0 to 10 (0= very uncomfortable, 5= neutral, 10=very comfortable).

1. How confident are you in the obstetrics ward?

2. How confident are you in being able to work as a team?

3. During the management of postpartum hemorrhage, how confident are you in your ability to delegate tasks?

4. When things are not going well on the obstetric ward, how confident are you in your ability to speak up?

5. When dealing with postpartum hemorrhage, how confident are you in your ability to act on directions from other members of healthcare team?

6. How confident are you in your ability to perform fundal massage?

7. During postpartum hemorrhage, how confident are you in your ability to place additional intravenous lines?

8. During postpartum hemorrhage, how confident are you in your ability to administer intravenous medications?

9. During postpartum hemorrhage, how confident are you in your ability to administer intramuscular medications?

10. How confident are you in your ability to verify correct blood products before administration?

11. How confident are you in your ability to recognize obstetric hemorrhage?

12. How confident are you in your ability to correctly treat postpartum hemorrhage?

13. In order to manage postpartum hemorrhage, how confident are you in your ability to choose appropriate drugs?

14. In order to manage postpartum hemorrhage, how confident are you in your ability to recall dosages and routes for drug administration?

15. During postpartum hemorrhage, how confident are you in your ability to estimate blood loss?

**Questions regarding the simulation:**

1. How long do you think the simulation lasted (not including the debriefing)?

2. Did time seem to speed up or slow down during the simulation?

a. No

b. Yes. How long did the simulation seem to take? ____________

3. Do you think mannequin-based simulation is an effective way to learn to care for a woman with postpartum hemorrhage?

4. Do you think screen-based simulation is an effective way to learn to care for a woman with postpartum hemorrhage?

5. Would you recommend this particular simulation as a way to learn about postpartum hemorrhage?

Now that you have finished this learning module, please give us your feedback on the software. Please mark the number corresponding to your response on a scale of 1 to 5 (1= strongly disagree, 2=disagree, 3=neutral, 4=agree, 5= strongly agree).

1. It is important to have access 24 hours a day, 7 days a week , 365 days a year

2. I would like to participate in the simulation on my own device (rather than being required to use a dedicated computer)

3. For each training session, it is important to have collaborators in the U.S.

4. I would like to be able to learn independently, without the need for other team players (I would like this training to be a single-player training)

5. I would like to continue learn in an environment with other human team players (I like this training as a multi-player training)

6. It is important to have immediate, in game feedback.

7. It is important to have feedback in a debrief with a live facilitator.

8. It is important to access this training on a mobile device.

9. It is important to have a live facilitator.

10. It is important for the software to give me immediate feedback.
